# Prevalence of perilymphatic fistula in patients with sudden-onset sensorineural hearing loss as diagnosed by Cochlin-tomoprotein (CTP) biomarker detection: its association with age, hearing severity, and treatment outcomes

**DOI:** 10.1007/s00405-023-08368-0

**Published:** 2023-12-21

**Authors:** Akira Sasaki, Tetsuo Ikezono, Han Matsuda, Ryuichiro Araki, Tomohiro Matsumura, Shiho Saitoh, Koichiro Wasano, Atsushi Matsubara

**Affiliations:** 1 Department of Otorhinolaryngology-Head and Neck Surgery, Hirosaki University Graduate School of Medicine, Hirosaki, Japan; 2 Faculty of Medicine, Department of Otorhinolaryngology, Saitama Medical University, Saitama, Japan; 3 Community Health Science Center, Saitama Medical University, Saitama, Japan; 4 Isotope Research Laboratory, Nippon Medical School, Tokyo, Japan; 5 Department of Otolaryngology-Head and Neck Surgery, Tokai University School of Medicine, Isehara, Japan

**Keywords:** Sudden-onset sensorineural hearing loss, Perilymphatic fistula, Cochlin-tomoprotein, Aging, Intratympanic dexamethasone therapy

## Abstract

**Purpose:**

To determine the prevalence of perilymphatic fistula (PLF) in sudden-onset sensorineural hearing loss (SSNHL) patients by employing the Cochlin-tomoprotein (CTP) detection test, a specific diagnostic marker for perilymph. We also analyzed the clinical characteristics associated with hearing outcomes in this cohort.

**Methods:**

A total of 74 eligible patients were prospectively enrolled. Following myringotomy, middle ear lavage (MEL) samples underwent the CTP test to identify perilymph leakage. Intratympanic dexamethasone (IT-DEX) therapy was administered, and hearing outcomes were assessed. Control groups comprised patients with chronic otitis media (*n* = 40) and non-inflammatory middle ears (*n* = 51) with concurrent MEL sample collection.

**Results:**

CTP was positive in 16 (22%) patients. No control samples showed positive results. Multiple regression analysis indicated that age and pre-treatment hearing levels significantly contributed to the CTP value. We found a positive correlation between CTP values, age, and pre-treatment pure-tone averages. Notably, CTP values in SSNHL cases aged 60 and above were significantly higher than in those below 60 years. Patients with positive CTP had significantly worse recovery rates after IT-DEX treatment.

**Conclusion:**

This study is the first prospective investigation demonstrating a positive relationship between CTP values, age, and hearing severity in SSNHL, indicating that PLF might be the essential cause of SSNHL, particularly in the elderly. Our findings suggest that IT-DEX may be less effective for PLF-associated SSNHL. Future research could reveal that PLF repair surgery is a viable treatment strategy for SSNHL. This study was registered under the UMIN Clinical Trials Registry (UMIN000010837) on 30/May/2013.

## Introduction

Sudden-onset sensorineural hearing loss (SSNHL) is a perplexing condition marked by rapid hearing loss with various potential underlying etiologies. Known causes account for nearly 10% of SSNHL cases, ranging from viral infections, such as mumps and varicella-zoster virus, to systemic illnesses like syphilis, exposure to ototoxic drugs and loud noises, autoimmune diseases, and even acoustic neuromas [1–4]. Another cause that has been implicated is perilymphatic fistula (PLF).

Treatment modalities for SSNHL are diverse, encompassing round and/or oval window patching surgery, which has been reported in numerous studies [5–11]. Many SSNHL patients have been treated with PLF repair surgery, as described by Hoch et al. who reported 51 cases over seven years, and Toth et al. who reported 85 cases over three years [10, 11]. However, the absence of a reliable biomarker for PLF has sparked a longstanding debate about the existence and surgical treatment of this condition.

With advances in proteomic analysis, we have identified an isoform of Cochlin, Cochlin-tomoprotein (CTP), as a perilymph-specific protein. The detection of CTP in the middle ear suggests the presence of perilymph leakage, with remarkably high diagnostic accuracy demonstrating a sensitivity of 86.4% and a specificity of 100%, which has profoundly changed the diagnosis landscape of PLF [12–16]. In these former studies, we have demonstrated the utility of this test in various neuro-otological diseases and conducted a nationwide survey of PLF/CTP detection.

The objective of this study is to ascertain the prevalence of PLF among SSNHL patients at our institution, to characterize its clinical features. Corticosteroid therapy is widely used to treat SSNHL; this study also aimed to evaluate the hearing outcomes of intratympanic dexamethasone (IT-DEX) treatment with reference to the CTP detection test.

## Materials and methods

### Study design and participants

We conducted a case–control study involving patients with SSNHL and control cases (described below) that were prospectively registered in the UMIN Clinical Trials Registry (UMIN000010837). The study spanned four years, from January 2013 to December 2016, and was conducted at the otorhinolaryngology clinics of Hirosaki University Hospital and Saitama Medical University Hospital. The study adhered to the ethical standards of the responsible committee on human experimentation and the Helsinki Declaration. The Committee of Medical Ethics of Hirosaki University Graduate School of Medicine (2012-195) and Saitama Medical University (13-086) approved the study protocol. Before undergoing any procedures, all participants provided written informed consent. For minors, consent was obtained from their guardians. The inclusion criteria required participants to have intact tympanic membranes, be 12 years or older, and have a more than three-month follow-up period. The study comprised two groups; SSNHL patients and control cases (Table 1).

**Table 1:** The demographic characteristics of the SSNHL patients and the control cases

	SSNHL	Control
No. of cases	74	91
Gender (male: female)	31:43	36:55
Age (median)	60.0	60.0

There were no significant differences in gender or age between these groups, as determined by Fisher’s exact test and Mann–Whitney *U*-test, respectively

### SSNHL patients

Sudden-onset sensorineural hearing loss (SSNHL) was defined as a sensorineural hearing loss of 30 dB or more across at least three contiguous audiometric frequencies within 72 h [4]. The inclusion criteria for IT-DEX therapy and pre- and post-treatment hearing ability assessment were determined using the pure-tone average (PTA), defined as the arithmetic mean of five frequencies (0.25, 0.5, 1, 2, and 4 kHz).

We performed pure-tone audiometry, laboratory tests including IgM and IgG antibodies to the varicella-zoster virus, syphilis tests, MRI scans, and pneumatic otoscopy for nystagmus, pressure-induced nystagmus, or vertigo exploration. We also collected medical histories, including any triggers or preceding traumatic or barotraumatic events leading to the onset of hearing loss and vestibular symptoms.

The Japanese PLF Study Group has recently classified the etiology of PLF into four categories, each linked to the origin of the condition (Table 2) [17]. It is essential to highlight that those cases classified as Category 1, which include those associated with direct labyrinthine trauma, middle and inner ear disorders of known etiology, and procedures involving middle and/or inner ear surgeries, are not typically classified as SSNHL. As such, these cases were not enrolled in this study. The SSNHL patients enrolled in this study were prospectively registered and categorized as below: Category 2 includes cases with barotraumas caused by external antecedent events; Category 3 includes cases with barotraumas caused by internal antecedent events; and Category 4 includes idiopathic cases with no apparent antecedent event.

**Table 2:** Categorization of PLF

*Category 1*
Linked to trauma, middle and/or inner ear diseases, surgeries
(1) a Direct labyrinthine trauma (stapes luxation, otic capsule fracture, etc.)
b Other trauma (head injury, body contusion, etc.)
(2) a Middle or inner ear diseases (cholesteatoma, tumor, anomaly, dehiscence, etc.)
b Iatrogenic (ear surgeries, medical treatments, etc.)
*Category 2*
Linked to barotrauma caused by antecedent events of external origin
(such as flying or diving)
*Category 3*
Linked to barotrauma caused by antecedent events of internal origin
(such as straining, sneezing, or coughing)
*Category 4*
Has no apparent antecedent event (idiopathic)
Note:”Spontaneous” should not be used

Exclusion criteria included bilateral SSNHL, known causes for acute hearing loss, such as inflammatory ear diseases, vestibular schwannoma, central nervous system disorders, or middle/inner ear anomalies identified by MRI/CT imaging, viral labyrinthitis, neurosyphilis, autoimmune disease by clinical symptoms and laboratory tests, exposure to ototoxic drugs, loud noise, trauma to the middle ear/temporal bone, bilateral progressive hearing loss, and other conditions deemed inappropriate for the trial (severe systemic illness, pregnancy, or lactation). We excluded patients with a PTA of less than 40 dB as these cases do not meet IT-DEX therapy criteria in our institution.

### Intratympanic dexamethasone (IT-DEX) therapy

All SSNHL patients underwent IT-DEX therapy as per our clinic protocol [18]. The patient lies in a supine position with the affected ear upward, 0.5 ml (4 mg/ml) of dexamethasone was injected through the perforation made by CO_2_ laser (OtoLAM; LUMENIS, Yokneam, Israel). The patient’s head is tilted 45 degrees away with the chin upward to bathe the round window membrane. The patient is instructed to remain in this position for 30 min without swallowing. Dexamethasone administration is performed on eight sequential days.

### Hearing outcome measurement

Hearing outcomes were evaluated at least three months after IT-DEX using a set of defined criteria. Complete recovery was defined as a study subject’s audiogram having one of the following two conditions: (1) the PTA on the affected side was 20 dB or less; (2) If there was hearing loss on the healthy side and the hearing level on the healthy side was determined to be stable, the PTA on the affected side recovered to within 10 dB of the healthy side’s PTA. These definitions were determined by the Ministry of Health, Labor and Welfare in Japan [19].

### Control cases

During the same study period, control samples were collected from patients undergoing tympanoplasty due to chronic otitis media with perforation (without active infection) (*N* = 40) and patients undergoing exploratory tympanotomy for conductive hearing loss (*N* = 25) or cochlear implantation (*N* = 26).

### CTP detection test

The CTP detection test involved collecting samples by lavaging the middle ear cavity with 0.3 ml of saline and retrieving the fluid, a procedure defined as middle ear lavage (MEL) [15]. In SSNHL patients, MEL was collected post-myringotomy using a CO_2_ laser. For the control cases, MEL was collected during surgery either through the perforation of the tympanic membrane or upon entering the middle ear without manipulating the ossicles or round window. All samples were subsequently centrifuged at 1250 g for one minute, and the supernatants were frozen and preserved at  – 80 °C. The samples were then sent to the pathology laboratory, SRL, Inc (Tokyo). The CTP detection test followed the standard operation protocol utilizing a human Cochlin-tomoprotein (hCTP) ELISA test with a polyclonal antibody. The cutoff criteria were 0.4 < CTP negative, 0.4≦CTP < 0.8 intermediate, 0.8≦CTP positive [20]. The lower detection limit was 0.2 ng/ml. If the measurement was below 0.2, the result was described as 0.2.

### Statistical analysis

A certified statistician (RA) conducted statistical analyses using IBM SPSS Statistics for Windows version 25.0 (IBM Corp., Armonk, NY, USA) and SAS JMP for Windows version 14.3.0 (SAS Institute, Inc., Cary, NC, USA). The results of individual tests and their associated *P*-values are presented in the report. A *P*-value of < 0.05 was defined as statistically significant for all tests. For multiple regression analysis, we used the variance inflation factor (VIF) to check for the presence of multicollinearity in the independent variables. A VIF cutoff of less than 5 was adopted.

## Results

**Fig. 1:**
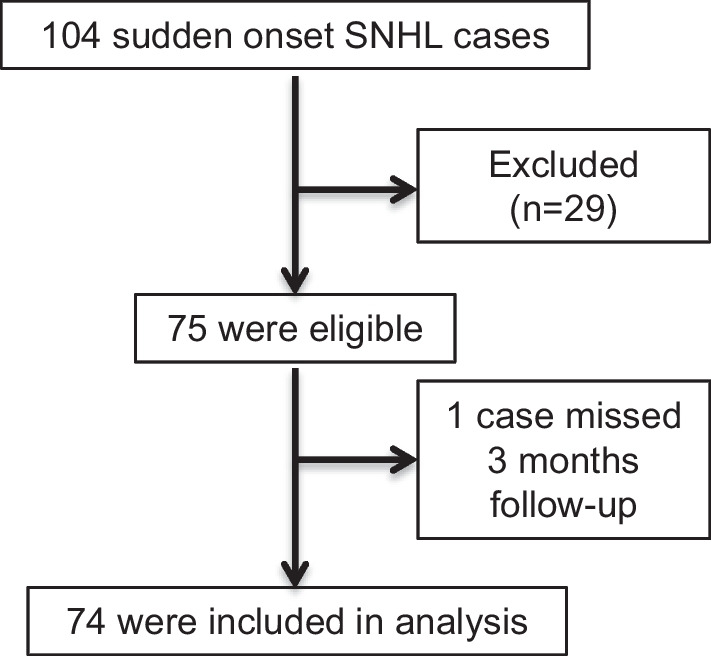
Flow diagram of study inclusion for SSNHL patients. During the trial period, 104 patients who were diagnosed with SSNHL visited our hospital for IT-DEX treatment. Out of these, 29 cases met the exclusion criteria. Initially, 75 patients were enrolled, but 1 patient missed the 3-month follow-up and was therefore excluded. Thus, leaving 74 eligible participants were included in the further analysis

Our hospital treated 104 patients diagnosed with SSNHL during the trial period using intratympanic dexamethasone injections. Out of these, 29 cases met the exclusion criteria as follows: 6 did not give consent; 2 were under 12 years of age; 1 was admitted after 28 days; 7 had mild hearing loss of less than 40 dB; 2 had acoustic neurinoma; 4 were diagnosed with a CNS disorder by MRI; 1 had neurosyphilis; 1 had polychondritis; 1 had chronic otitis media; and 4 had SNHL in the contralateral ear. Initially, 75 participants were enrolled. However, one participant did not complete the 3-month follow-up and was subsequently excluded. Thus, 30 cases were excluded, leaving 74 eligible participants for the study. A flow diagram detailing the inclusion of SSNHL patients in the study can be found in Fig. 1.

The demographics of the participants in SSNHL patients (74 cases) and control cases (91 cases) are outlined in Table 1. There were no significant differences in gender or age between these groups, as determined by Fisher’s exact test (*P* = 0.87) and Mann–Whitney *U*-test (*P* = 0.85), respectively.

The enrolled patients in this study were categorized into four groups based on criteria linked to the origin of their condition (Tables 2, 3). Category 2: Cases with barotraumas caused by external antecedent events, comprising 3 cases. Category 3: Cases with barotraumas caused by internal antecedent events, comprising 15 cases. Category 4: Idiopathic cases with no apparent antecedent event, comprising 56 cases. None of the three cases in Category 2 were CTP positive, whereas 5 of the 15 cases in Category 3 (33%) and 11 of the 56 cases in Category 4 (20%) were CTP positive. There was no relationship between the CTP-positive ratio and the categorization (Fisher–Freeman–Halton test, *P* = 0.33). 16 out of the 74 cases were CTP positive (22%).

**Table 3:** Categorization of patients and corresponding CTP test results

Category	Negative CTP < 0.4	Intermediate 0.4 ≤ CTP < 0.8	Positive 0.8 ≤ CTP	Total
2 Barotrauma of external origin	3 (100%)	0 (0%)	0 (0%)	3
3 Barotrauma of internal origin	5 (33%)	5 (33%)	5 (33%)	15
4 Idiopathic	23 (41%)	22 (39%)	11 (20%)	56
Total	31 (42%)	27 (36%)	16 (22%)	74

Data are number (percentage). There was no relationship between the CTP-positive ratio and the categorization (Fisher–Freeman–Halton test)

Multiple regression analysis was then performed to identify factors influencing the CTP value (Table 4). The dependent variable was the CTP value. Independent continuous variables included age, days from onset, and pre-treatment PTA. Binary variables were gender, preceding events (with or without identifiable antecedent events), and vestibular symptoms (vertigo, dizziness, disequilibrium). A significant positive correlation was found between age and CTP value, indicating that cases with a higher age tend to show higher CTP values. Additionally, a significant positive correlation was identified between pre-treatment PTA and CTP value, suggesting that a higher PTA is associated with a higher CTP value. No significant influence on CTP value was observed from the number of days from onset, the presence of preceding events, or the presence of vestibular symptoms. The VIF was used to check for the presence of multicollinearity in the independent variables. The VIF of all independent variables were ranged 1.061 to 1.316, and were considered acceptable. It is not necessary to consider multicollinearity among the independent variables.

**Table 4:** Multiple regression analysis for factors to influence on CTP value

	Standardized regression coefficient	*P*-value	Variance inflation factor	Adjusted *R*^2^
				0.172
Age	0.269	0.02	1.066	
Gender	0.225	0.06	1.200	
Days from onset	0.091	0.42	1.105	
Preceding events	0.154	0.16	1.061	
Pre-treatment PTA	0.313	0.01	1.316	
Vestibular symptoms	0.019	0.87	1.227	

There were significant positive correlations between age and CTP value, and pre-treatment PTA and CTP value

**Fig. 2:**
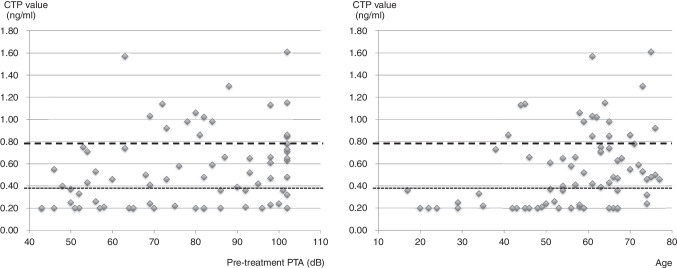
2-1 Relationship between CTP values and pre-treatment PTA. CTP values for the enrolled 74 SSNHL patients were arranged by corresponding pre-treatment PTA. CTP values ranging from 0.20 to 1.61. A weak but significant positive correlation between pre-treatment PTA and CTP values was recognized (Pearson’s correlation coefficient, *r* = 0.287, *P* = 0.01). The cutoff values were shown as dotted and dashed lines; below 0.4 was CTP negative; between 0.4 and 0.8 were intermediate; and more than 0.8 was CTP positive. 2-2 Relationship between CTP values and age. CTP values for the enrolled 74 SSNHL patients were arranged in accordance with corresponding age. A significant positive moderate correlation between age and CTP values was recognized (Pearson’s correlation coefficient, *r* = 0.327, *P* = 0.004). The cutoff values were shown as dotted and dashed lines; below 0.4 was CTP negative; between 0.4 and 0.8 were intermediate; and more than 0.8 was CTP positive

Statistical analysis revealed a weak but significant positive correlation between pre-treatment PTA and CTP values ranging from 0.20 to 1.61 (Pearson’s correlation coefficient, *r* = 0.287, *P* = 0.01, Fig. 2-1). A moderate positive correlation was also found between age and CTP values (Pearson’s correlation coefficient, *r* = 0.327, *P* = 0.004, Fig. 2-2). This suggests that both higher PTA and older age are associated with higher CTP values. There was no statistically significant correlation between age and PTA values (Pearson’s correlation coefficient, *r* = 0.039, *P* = 0.74).

**Fig. 3:**
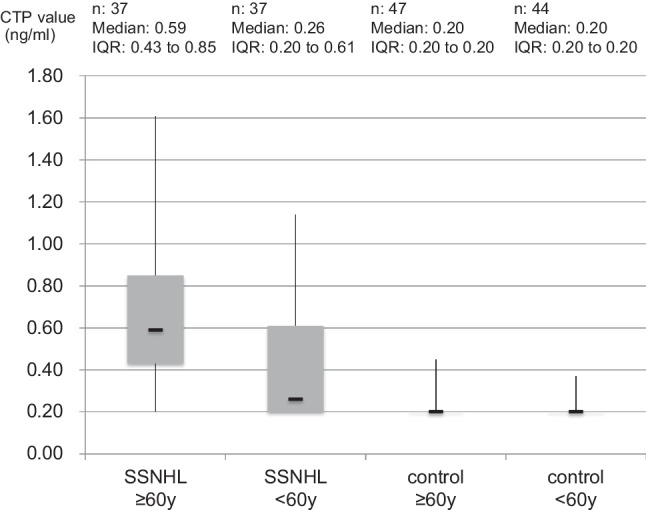
A box-and-whisker plot for CTP values of SSNHL patients and control cases. SSNHL patients and control cases were further subdivided by age 60. Plots showing the median, interquartile ranges (IQRs, i.e., the differences between the 25% and 75% percentiles), and minimum/maximum values of CTP. The numbers of cases in each group and CTP values (median, IQRs, and maximum and minimum) were shown in the graph

SSNHL patients and control cases were further subdivided by the age of 60. In the SSNHL patient group, there were 37 cases aged 60 and above and 37 cases below 60. In the control group, there were 47 cases aged 60 and above and 44 cases below 60. Figure 3 shows a box-and-whisker plot of CTP values. The number of cases in each group and CTP values (including median and interquartile ranges (IQRs)) are indicated in the graph. In the control group, 88 cases (97%) tested negative for CTP. This includes 80 cases where CTP values were equal to or below the detection limit of 0.2 mg/ml. Only three samples were classified as intermediate, and none tested positive. We utilized two-way ANOVA followed by the Steel–Dwass test for multiple comparisons to statistically determine whether the disease condition (SSNHL) and age affect CTP values compared to the control group. A statistically significant synergic interaction was observed between these two factors (*P* = 0.002). This finding led us to conduct the Steel–Dwass test for multiple comparisons across the four groups. We found that the CTP values in SSNHL patients aged 60 and above were significantly higher than those in SSNHL patients under 60 (*P* = 0.009). However, there was no significant difference in CTP values between control cases aged 60 and above and control cases under 60 years of age (*P* = 0.80). These results indicate that in patients with SSNHL, age appears to impact CTP values, with higher values noted in those aged 60 and above. However, age did not affect CTP values in the control group.

We aimed to discern differences in treatment efficacy between CTP-positive and -negative cases. For our statistical analysis, we omitted intermediate cases, focusing instead on binary data. In the cohort of CTP-negative cases, we observed complete recovery in 45% (14 out of 31) of the patients, while 55% (17 out of 31) exhibited varying degrees of non-complete recovery. Conversely, in the CTP-positive group, only 13% (2 out of 16) demonstrated complete recovery, whereas 88% (14 out of 16) showed non-complete recovery. These results are detailed in Table 5. We then conducted a statistical comparison to assess whether the complete recovery rate correlated with CTP values. We found a statistically significant disparity in complete recovery rates between the CTP-positive and CTP-negative cases (Fisher’s exact test, *P* = 0.049).

**Table 5:** Complete recovery rate and CTP-positive rate

	Negative CTP < 0.4	Positive 0.8 ≤ CTP	Total
Complete recovery	14 (45%)	2 (13%)	16 (34%)
Non-complete recovery	17 (55%)	14 (88%)	31 (66%)
Total	31	16	47

Data are number (percentage). There was a significant difference in complete recovery rates between CTP-positive and CTP-negative cases (Fisher’s exact test, *P* = 0.049)

Table 6 presents the result of multiple logistic regression analysis to identify the factors impacting the complete recovery in both CTP-positive and -negative cases. The dependent variable was the complete recovery or not. Independent variables included were CTP positive or negative, age, gender, days from onset, presence of preceding events, pre-treatment PTA, and vestibular symptoms. Out of all the factors evaluated, pre-treatment PTA was the only variable found to significantly influence the complete recovery rate (*P* = 0.001, odds ratio [OR] 0.921, 95% confidence interval [CI] 0.863–0.981).

**Table 6:** Multiple logistic regression analysis for factors to influence the complete recovery

	*P*-value	Odds ratio (95% confidence interval)
CTP positive	0.12	0.175 (0.020–1.545)
Age	0.53	0.977 (0.907–1.051)
Gender	0.10	7.696 (0.656–90.326)
Days from onset	0.45	0.948 (0.825–1.089)
Preceding events	0.63	0.561 (0.054–5.777)
Pre-treatment PTA	0.01	0.921 (0.863–0.981)
Vestibular symptoms	0.59	0.602 (0.098–3.710)

Pre-treatment PTA was the only factor found to significantly influence the complete recovery

## Discussion

PLF has been suggested as a cause for SSNHL [1, 5], but the lack of an appropriate biomarker has made this hypothesis controversial. In this study, we utilized a modern classification of PLF (Table 2) along with the perilymph-specific biomarker CTP and prospectively registered 74 SSNHL cases. Among these cases, 18 had antecedent barotraumatic events (external/internal), classified as categories 2 and 3, while the remaining 56 cases were categorized as idiopathic (category 4). Due to the prospective nature of our study and the enrollment of patients in a systematic manner, there are minimal missing values in the dataset.

Following our hospital’s protocol, SSNHL patients are administered IT-DEX. The CTP detection test can be conveniently performed during this treatment using the tympanotomy hole created for the dexamethasone injection. Out of the 74 cases analyzed, 16 tested positive for CTP, resulting in a total positive ratio of 22%. The detailed distribution of the positive ratio within each category is presented in Table 3. Interestingly, the categorization did not significantly impact the CTP-positive ratio, as determined by the Fisher–Freeman–Halton test. Before initiating this clinical trial, we anticipated a higher incidence of CTP-positive cases among categories 2 and 3, linked to barotraumatic antecedent events. However, only age and pre-treatment PTA were found to influence the CTP value.

Additionally, we conducted a multiple regression analysis to identify factors influencing CTP value (Table 4). We observed that SSNHL cases with worse pre-treatment PTA tended to have elevated CTP values (Fig. 2-1) (*r* = 0.287). This finding is logically consistent as perilymph leakage could cause more extensive damage to cochlear function compared to other causes of SSNHL. Age also emerged as a significant factor influencing CTP values in SSNHL cases. We identified a moderate correlation between age and CTP values through statistical association analysis, as depicted in Fig. 2-2 (*r* = 0.327). Notably, there were no CTP-positive cases among patients younger than 40 years old.

Further, it is essential to consider potential routes of perilymph leakage, including through the round window. Carpenter et al. investigated the morphological features of 14 human round window membranes (RWMs). They discovered that while the average thickness of 70 microns remained consistent with age, the connective tissue in older individuals exhibited a looser arrangement with increased ground substance. The elastic fibers became thicker, fibroblast nuclei enlarged, rounder, and displayed irregular extensions [21]. Animal studies revealed that the RWMs of aged mice were thinner than younger mice [22]. These findings suggest that age-related morphological changes in the RWM may contribute to its weakening [21]. Other structures, such as the stapes annular ligament [23] and the fistula ante fenestram (FAF) [11], might be impacted by aging. However, these require further histological investigation.

To elucidate whether the age-dependent increase in CTP values is related to the pathology of SSNHL, we also analyzed CTP values in 91 age-matched control cases (Fig. 3). It was observed that SSNHL patients aged 60 years and above exhibited significantly higher CTP values compared to those below 60 years (*P* = 0.009) (Fig. 3). However, no significant age-related differences in CTP values were found among the control cases (*P* = 0.80). These findings imply that the increased CTP values in older SSNHL patients are likely attributable to the pathology of the condition rather than physiological aging. This novel observation could shed light on certain epidemiological characteristics of SSNHL.

Past epidemiological studies have indicated an age-related increase in the incidence of SSNHL [24]. In a nationwide Japanese epidemiological survey of SSNHL, we similarly observed an increase in cases with age, peaking in the 60 s [25]. Historically, this increase was attributed to vascular complications such as atherosclerosis, thrombosis, hyperlipidemia, diabetes mellitus, heart disease, and stroke [3, 25, 26]. However, our current study suggests that an increase in the incidence of PLF could also be a contributing factor.

It is important to note this study is an exploratory case–control study, and the direct causal relationship between CTP values, age, and pre-treatment PTA is unknown. However, it has been revealed that PLF may be present in the elderly population and those with high pre-treatment PTA values. We are currently planning confirmatory research to investigate these associations further.

Despite previous reports suggesting that vestibular symptoms indicate a higher likelihood of PLF, our multiple regression analysis did not find any significant influence of vestibular symptoms on CTP values (Table 4).

All participants in this study received IT-DEX treatment. After a three-month follow-up, 2 out of 16 CTP-positive cases (13%) exhibited complete recovery, in contrast to 14 out of 31 CTP-negative cases (45%) (Table 5). This significant difference in recovery rates suggests that IT-DEX might be more effective in treating SSNHL in patients without elevated CTP values, as Fisher’s exact test determined.

Previous studies have shown that pre-treatment PTA significantly influences the recovery rate of SSNHL [25]. This finding is consistent with our multiple logistic regression analysis, which indicated that pre-treatment PTA was the only factor significantly affecting complete recovery rates (Table 6). Why CTP values were not identified as a factor influencing the complete recovery rate remains unclear. However, we require a larger sample size to discern the relationship with more precision.

Glucocorticoids (GCs) are widely used to suppress inflammation in various diseases, including hearing loss [1, 3, 25, 27]. However, GCs have known side effects, including impaired wound healing in various tissues [28]. While the side effects of GCs in the treatment of inner ear dysfunction are not fully understood, there is a possibility that they may produce adverse outcomes in PLF-induced SSNHL.

Our earlier research indicated that IT-DEX exhibited a high response and cure rate as a standalone first-line treatment for SSNHL patients [18]. However, a recent Cochrane Database of Systematic Reviews review suggested that intratympanic corticosteroids may have limited or no benefit compared to systemic corticosteroids for primary treatment [29]. This warrants a reevaluation of our treatment approach for SSNHL and PLF.

PLF repair surgery is also a treatment option for SSNHL and is practiced by many otologic surgeons [5–10]. A recent study by Thomas et al. reported that sealing both the round and oval windows in 136 SSNHL cases resulted in complete recovery or substantial improvement in 38 cases (28%), with 105 subjects (77%) experiencing an improvement of more than 10 dB [9]. While these studies vary in diagnostic criteria, surgical timing, patching materials, and outcome measures, patients generally responded positively to surgical intervention.

This study presents several limitations. An exploratory investigation was conducted to ascertain the prevalence of perilymphatic fistulas within SSNHL patients over the study period. The findings of this investigation have prompted us to explore the design of a subsequent clinical study. A sub-analysis was undertaken to investigate the correlation between age, CTP test results, and the severity of pre-treatment PTA. This analysis yielded significant scientific insights, leading to the decision to report these findings within the current study. Nonetheless, the direct causal relationship between CTP values, age, and pre-treatment PTA remains to be clarified. Our key finding is that older individuals and those with high pre-treatment PTA values may harbor perilymph fistulas. This observation forms the basis of our planned future confirmatory studies specifically targeting these demographic groups.

## Conclusion

In this study, we conducted a case–control study of 74 SSNHL patients and 91 controls, revealing that 22% of the cases were CTP positive, suggesting PLF is the essential cause of SSNHL, particularly among the elderly population with more severe hearing loss. One of the standard therapies for SSNHL, intratympanic dexamethasone injection, is less effective for PLF-associated SSNHL. Additionally, the treatment outcomes from our study suggest that one of the standard therapies for SSNHL, IT-DEX therapy, may be less efficacious for cases with PLF and may not be recommended for elderly patients. These findings hold significant implications for enhancing our understanding of the etiology of SSNHL and guiding the selection of treatment strategies for this condition. Future research may uncover the potential of PLF repair surgery as a viable therapeutic option for SSNHL.

## Data Availability

The datasets during the current study are available from the corresponding author (TI) on reasonable request.
